# Observation and theoretical calculations of voltage-induced large magnetocapacitance beyond 330% in MgO-based magnetic tunnel junctions

**DOI:** 10.1038/s41598-021-93226-4

**Published:** 2021-07-12

**Authors:** Kentaro Ogata, Yusuke Nakayama, Gang Xiao, Hideo Kaiju

**Affiliations:** 1 Faculty of Science and Technology, Keio University, Yokohama, Kanagawa 223-8522 Japan; 2 Department of Physics, Brown University, Providence, RI 02912 USA; 3 Center for Spintronics Research Network, Keio University, Yokohama, Kanagawa 223-8522 Japan

**Keywords:** Spintronics, Magnetic devices

## Abstract

Magnetic tunnel junctions (MTJs) in the field of spintronics have received enormous attention owing to their fascinating spin phenomena for fundamental physics and potential applications. MTJs exhibit a large tunnel magnetoresistance (TMR) at room temperature. However, TMR depends strongly on the bias voltage, which reduces the magnitude of TMR. On the other hand, tunnel magnetocapacitance (TMC), which has also been observed in MTJs, can be increased when subjecting to a biasing voltage, thus exhibiting one of the most interesting spin phenomena. Here we report a large voltage-induced TMC beyond 330% in MgO-based MTJs, which is the largest value ever reported for MTJs. The voltage dependence and frequency characteristics of TMC can be explained by the newly proposed Debye-Fröhlich model using Zhang-sigmoid theory, parabolic barrier approximation, and spin-dependent drift diffusion model. Moreover, we predict that the voltage-induced TMC ratio could reach over 3000% in MTJs. It is a reality now that MTJs can be used as capacitors that are small in size, broadly ranged in frequencies and controllable by a voltage. Our theoretical and experimental findings provide a deeper understanding on the exact mechanism of voltage-induced AC spin transports in spintronic devices. Our research may open new avenues to the development of spintronics applications, such as highly sensitive magnetic sensors, high performance non-volatile memories, multi-functional spin logic devices, voltage controlled electronic components, and energy storage devices.

## Introduction

Magnetic tunnel junctions (MTJs), which consist of a thin insulating layer sandwiched by two ferromagnetic (FM) layers, are among the key devices in the field of spintronics. Owing to the large tunneling magnetoresistance (TMR) at room temperature, MTJs have been utilized for hard disk drives, magnetic random access memories (MRAMs), and highly sensitive magnetic sensors^1–4^. Other applications include strain-gauge sensors^5,6^, MEMS microphone^7,8^, flexible sensors^9,10^, nuclear magnetic resonance (NMR) signal detectors^11–13^, and neuromorphic computing devices^14,15^. MTJs have been wlidely used in various fields of electronic devices.

Engineering the robustness of TMR against a bias voltage is one of the most important issues for the achievement of high magnetic sensitivity in MTJs^16–18^. As is well known, TMR decreases with increasing bias voltage, which lowers sensitivity^19,20^. This reduction is attributed to hot-electron properties in the anti-parallel state of the bi-ferromagnetic (FM) layers^19^. For example, zero-bias TMR of 377% and 89% are observed in MgO- and AlO-based MTJs using Co_70_Fe_30_ FM layers, and it decreases to 75% and 20%, respectively, at a bias voltage of 0.5 V^21^. The improvement of bias-reduced TMR has been reported in MTJs with a small lattice mismatch (a few percent) between the FM and the insulating layers^22–27^. Zero-bias TMR is 92% in fully epitaxial Fe/MgO/GaO_x_/Fe MTJs, and the half biasing voltage *V*_1/2_, where the TMR is halved, can be raised to 0.5 V^22^. Moreover, TMR is 117% near zero bias and *V*_1/2_ is relatively large, ranging from 1.0 to 1.3 V in lattice-matched Fe/spinel MgAl_2_O_4_/Fe MTJs^23–26^. Further improvement has been reported. TMR is 240% near zero bias, and it keeps a large value of 180% at 0.5 V in MgAl_2_O_4_-based MTJs^27^. Nevertheless, a bias voltage always reduces TMR, which remains an essential problem in the applications of MTJs.

As a complementary effect to TMR, tunneling magnetocapacitance (TMC) is now actively being investigated due to its unique properties, such as high magnetic sensitivity, thermal stability, and robustness against bias voltage^28–32^. Since the magnetocapacitance (MC) effect is observed in a system with broken time-reversal and space-inversion symmetry, the research of TMC is of particular importance for both practical applications and for fundamental physics^28^. TMC is larger than TMR at a specific frequency, and it can be well accounted for by the Debye–Frölich (DF) model^29^. The maximum TMC reported previously is 155% in MgO-based MTJs featured with a TMR of 100%. In this case, the DF model calculation predicts a maximum TMC of 1000%. As a result of the presence of spin capacitance^30^, TMC has been shown to be temperature independent. Perhaps more significantly, TMC is more robust against biasing than TMR. The *V*_1/2_ of TMC is almost twice as high as that of TMR in MgO-based MTJs^31^. Our recent work also demonstrates that TMC actually slightly increases from 98% up to 102% upon biasing, which correspondingly causes the TMR to decay from 100% to 50%^32^.

In this work, we report a new phenomenon of bias induced *doubling* of the magnitude of TMC in an MTJ system based on a stack of Co_40_Fe_40_B_20_/MgO/Co_40_Fe_40_B_20_. We have observed a maximum TMC value of 332% under a bias voltage, which is the largest TMC ever reported for MTJs. There is an excellent agreement between theory and experimental results for the TMC in the entire voltage regions at each frequency using DF model incorporating a parabolic barrier approximation (PBA), spin-dependent drift diffusion (SDD) model, and Zhang-sigmoid theory. Based on our calculations, we predict that the voltage-induced TMC ratio could reach 3000% in MTJs, representing a dramatic spintronics effect which can potentially benefit applications in various areas.

## Results and discussion

### Device structure and measurement of TMC and TMR

We have prepared MTJ multilayer stack structures using a high vacuum magnetron sputtering system with a base pressure of 2 × 10^–8^ Torr, with the following layer sequence: SiO_2_/Ta(5)/Co_50_Fe_50_(2)/IrMn(15)/Co_50_Fe_50_(2)/Ru(0.8)/Co_40_Fe_40_B_20_(3)/MgO(2)/Co_40_Fe_40_B_20_(3)/Contact layer (numbers referred to thickness in nm). Details of the device fabrication procedure are described in the Experimental Section. Using standard photolithography, we have patterned the multilayer MTJ stacks into a junction area of 1800 μm^2^ with an elliptical shape with physical Ar ion-milling and SiO_2_ insulation overlayer. The frequency characteristics and the bias voltage dependence of the TMC and TMR for MTJs were measured by an AC four-probe method at room temperature. The AC voltage was set to 2.6 mV_rms_. The magnetic field was applied along the magnetic easy-axis direction to 1.4 kOe.

### Modeling of TMC

**Figure 1:**
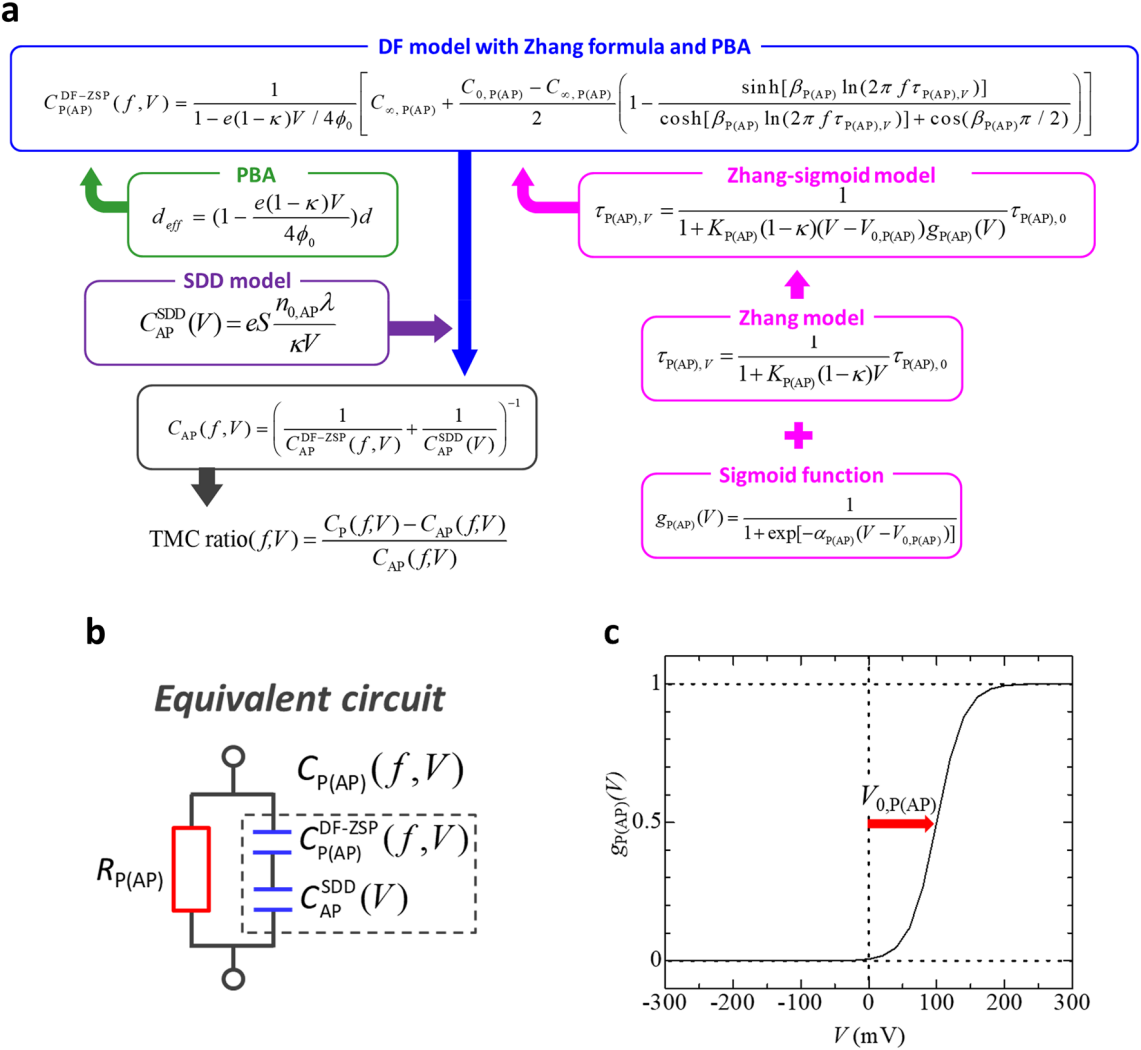
Modeling of TMC. (**a**) Debye-Fröhlich (DF) model incorporating Zhang-sigmoid model, parabolic barrier approximation (PBA), and spin-dependent drift–diffusion (SDD) model. (**b**) Equivalent circuit model of MTJs. (**c**) Sigmoid function.

Figure 1a shows the schematic of calculation procedure for the analysis of the frequency characteristics and bias dependence of TMC. The calculation is performed based on a newly proposed DF model using Zhang-sigmoid model in addition to the conventional models of PBA and SDD. Here, we describe each model in detail. The DF model describes the dielectric dispersion of electric dipoles, and it can be applied to various systems such as insulators, semiconductors, metals, or organic molecular liquids. The DF model can also be used as tools for explanation of frequency characteristics of magnetocapacitance effect^33–35^. On the basis of the model, the capacitance ${C}_{{\rm P}\left(\text{AP}\right)}^{{\rm DF}}(f)$ as a function of frequency *f* for the Parallel (Antiparallel), P(AP), configuration in MTJs can be expressed by

1 $$C_{{{\rm P(AP)}}}^{{{\rm DF}}} (f) = \text{Re} \left[ {C_{{\infty ,\,\text{P(AP)}}} + \frac{{C_{{0,\,\text{P(AP)}}} - C_{{\infty ,\,\text{P(AP)}}} }}{{1 + (i2\pi {\kern 1pt} f\tau _{{{\rm P(AP)}}} )^{{\beta _{{{\rm P(AP})}} }} }}} \right],$$

where *C*_∞,P(AP)_ and *C*_0,P(AP)_ are the high-frequency and DC capacitances, *τ*_P(AP)_ is the relaxation time, and *β*_P(AP)_ is the exponent showing the distribution of relaxation time, respectively, for the P(AP) configuration. The equivalent circuit is shown in Fig. 1b. By calculating Eq. (1), we can obtain

2 $$C_{{{\rm P(AP)}}}^{{{\rm DF}}} (f) = C_{{\infty ,\text{P(AP)}}} + \frac{{C_{{0,\text{P(AP)}}} - C_{{\infty ,\text{P(AP)}}} }}{2}\left[ {1 - \frac{{\sinh [\beta _{{{\rm P(AP)}}} \ln (2\pi {\kern 1pt} f\tau _{{{\rm P(AP)}}} )]}}{{\cosh [\beta _{{{\rm P(AP)}}} \ln (2\pi {\kern 1pt} f\tau _{{{\rm P(AP)}}} )] + \cos (\beta _{{{\rm P(AP)}}} \pi /2)}}} \right].$$

The relation between *τ*_P_ and *τ*_AP_ in FM/insulator/FM is given by

3 $$\tau _{{{\rm AP}}} = \frac{{1 + P_{{{\rm TMC}}}^{2} }}{{1 - P_{{{\rm TMC}}}^{2} }}\tau _{{\rm P}} ,$$

where *P*_TMC_ is the spin polarization, contributing to TMC, inside the FM layer^29,36^. Using these formulas, we can find frequency characteristics of TMC ratio, defined by

4 $$\text{TMC}(f) = \frac{{C_{{\rm P}}^{{{\rm DF}}} (f) - C_{{{\rm AP}}}^{{{\rm DF}}} (f)}}{{C_{{{\rm AP}}}^{{{\rm DF}}} (f)}}.$$

Then, we incorporate Zhang model to calculate TMC under bias voltage. According to Zhang model^19^, the conductance can be expressed by *G*_P(AP)*,V*_ = *G*_P(AP),0_ (1 + *K*_P(AP)_*V*), where *G*_P(AP),0_ is a conductance at zero bias in the P(AP) configuration and *K*_P(AP)_ is parameter determined by Curie temperatures of FM layers, the density of states of itinerant electrons in FM layers_,_ and direct and spin-dependent transfers and spin quantum number within the framework of the transfer Hamiltonian in the system of FM/insulator/FM. Moreover, we introduce sigmoid function into Zhang model to express the weighting of the applied voltage. The sigmoid function is expressed by

5 $$g_{{{\rm P(AP)}}} (V) = \frac{1}{{1 + \exp [ - \alpha _{{{\rm P(AP)}}} (V - V_{{0,\text{P(AP})}} )]}},$$

where *V*_0,P(AP)_ indicates the voltage at which the value of the sigmoid function becomes 0.5 in the P(AP) configuration as shown in Fig. 1c. *α*_P(AP)_ is the constant parameter, indicating the broadening of the sigmoid function, in the P(AP) configuration. Here, we describe the physical picture behind the sigmoid function. By applying a voltage and approaching *V*_0,P(AP)_, since the electrons gain energy, the spin flip is promoted. In the AP configuration, the spin accumulation occurs at the FM/insulator. Therefore, the voltage, contributed to the DF-modelled dynamic capacitance, in the AP configuration is smaller than that in the P configuration. For this reason, spin flip is more likely to occur in the P configuration than in the AP configuration at low voltages. This corresponds to the relationship of *V*_0,P_ < *V*_0,AP_ in sigmoid function. This means that the sigmoid function can express the spin-flip voltage. A large difference between *V*_0,P_ and *V*_0,AP_ could contribute the enhancement of TMC with respect to the voltage. The combination with Zhang model and sigmoid function provides the relaxation time *τ*_P(AP),*V*_ with applied voltage, which can be written by

6 $$\tau _{{{\rm P(AP)},\;V}} = \frac{1}{{1 + K_{{{\rm P(AP)}}} (1 - \kappa )(V - V_{{0,\text{P(AP)}}} )g(V)_{{{\rm P(AP)}}} }}\tau _{{{\rm P(AP)},\;0}} ,$$

where *τ*_P(AP),0_ is the relaxation time at zero bias voltage in the P(AP) configuration, and κ is an adjustable positive parameter of much smaller than 1.0. Equation (6) is called Zhang-sigmoid model. The relaxation time *τ*_P(AP),*V*_ in Eq. (6) should be used as the replacement of *τ*_P(AP)_ in Eq. (2) under the application of bias voltage.

PBA model is taken into account to describe the bias voltage dependence of the effective barrier thickness. The potential profile in the barrier is based on parabolic function, which is often used as an approximation for tunneling process, such as ac nonmagnetic and magnetic tunneling transport^37,38^. In this PBA, the potential function $\phi \left( u \right) = 4\phi _{0} (1 - u)u + eVu,$ where $u = x/d$ is the reduced spatial variable, $x$ is the distance from the surface of the one side electrode, $d$ is the barrier thickness, $\phi _{0}$ is the barrier height in the absence of the bias voltage and *e* is the electron charge. The solution of $\phi (u) = eV$ is $u_{1} = eV/4\phi _{0}$ and $u_{2} = 1$ (for $u_{1} < u_{2}$), the effective barrier thickness *d*_*eff*_ can be represented by

7 $$d_{{eff}} = (1 - \frac{{e(1 - \kappa )V}}{{4\phi _{0} }})d,$$

The calculation of $C_{{\rm AP}}(f,V)$ is performed using the SDD model in addition to DF model combined with PBA and Zhang-sigmoid model. SDD model illustrates that the accumulation of minority spins and the depletion of majority spins occur at the interface between the ferromagnetic layer and insulating layer in the AP configuration^37^. The spin accumulation gives rise to the creation of tiny screening charge dipoles, which act as an additional serial capacitance. This capacitance is called spin capacitance^37^, which can be represented by

8 $$C_{{{\rm AP}}}^{{{\rm SDD}}} (V) = eS\frac{{n_{{0,\,\text{AP}}} \lambda }}{{\kappa V}},$$

where *S* is a junction area, $n_{{0,{\rm AP}}}$ is a screening charge density and *λ* is a characteristic screening length at the interface in the AP configuration. Since this screening charge acts as a serial capacitance, the capacitance ${{C}}_{\text{AP}}({f}, V)$ under the applied DC voltage *V* in the AP configuration,

9 $$C_{{\text{AP}}} (f,V) = \left( {\frac{1}{{C_{{\text{AP}}}^{{{\rm DF} - \text{ZSP}}} (f,V)}} + \frac{1}{{C_{{\text{AP}}}^{{{\rm SDD}}} (V)}}} \right)^{{ - 1}} ,$$

where the capacitance ${C}_{{\rm AP}}^{{\rm DF-ZSP}}$($f, V$) based on the DF model combined with Zhang-sigmoid model and PBA in the AP configuration is represented by

10 $$C_{{\text{AP}}}^{{{\rm DF} - \text{ZSP}}} (f,V) = \frac{1}{{1 - e(1 - \kappa )V/4\varphi _{0} }}\left[ {C_{{\infty ,\;\text{AP}}} + \frac{{C_{{0,\;\text{AP}}} - C_{{\infty ,\;\text{AP}}} }}{2}\left( {1 - \frac{{\sinh [\beta _{{\text{AP}}} \ln (2\pi {\kern 1pt} f\tau _{{\text{AP},\,V}} )]}}{{\cosh [\beta _{{\text{AP}}} \ln (2\pi {\kern 1pt} f\tau _{{\text{AP},\,V}} )] + \cos (\beta _{{\text{AP}}} \pi /2)}}} \right)} \right].$$

### TMC and TMR under no bias voltage

**Figure 2:**
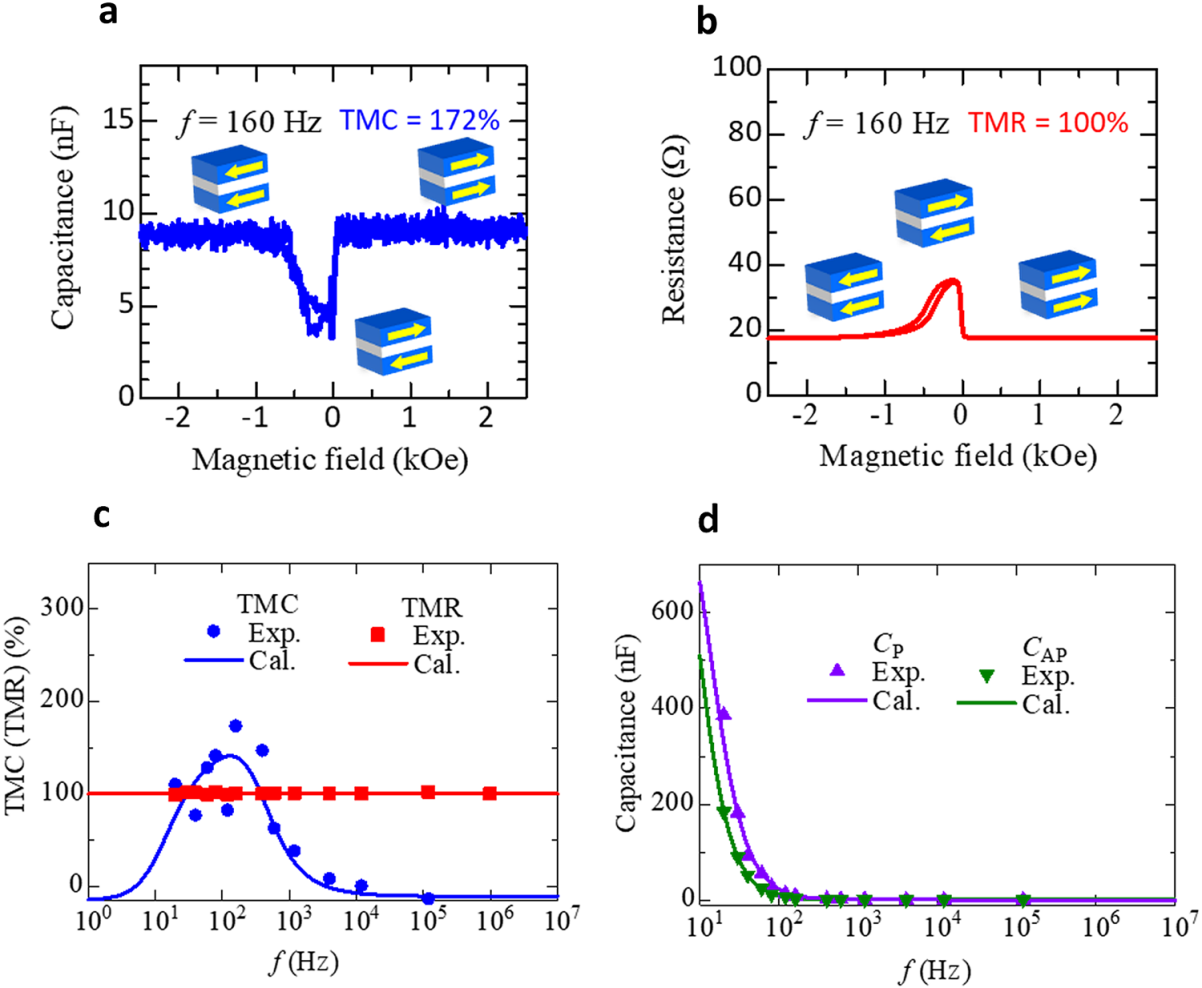
TMC and TMR at zero bias voltage. (**a**) TMC and (**b**) TMR transfer curves of an MgO-based MTJ sample at a frequency of 160 Hz. (**c**) Frequency dependence of TMC and TMR, experimental data and model calculations. Note the strong frequency dependence of TMC and independence of TMR. (**d**) The capacitance *C*_P(AP)_ versus frequency in the P(AP) configuration. The solid points are experimental data and the solid lines represent the calculation results based on Debye-Fröhlich model as described by Eq. (2).

Figure 2a, b shows the TMC and TMR curves at 160 Hz. The DC applied voltage is 0 V. Clear TMC and TMR are observed, i.e., and *C*_P_ > *C*_AP_ and *R*_P_ < *R*_AP._

TMC and TMR ratios are 172% and 100%, respectively, at room temperature. Figure 2c, d shows the frequency characteristics of TMC, TMR and *C*_P(AP)_. We calculated the frequency characteristics of the TMC and *C*_P(AP)_ using Eqs. (2)–(4). The calculation results of TMC and *C*_P(AP)_ under no bias voltage fit to the experimental data well. The calculation was performed using the following parameters: *C*_∞,P(AP)_ = 0.80 (0.90) nF, *C*_0,P(AP)_ = 1037 (1221) nF, *β*_P(AP)_ = 0.986 (0.999), *τ*_P_ = 0.0118 s and *P*_TMC_ = 0.477. According to the fitting result, *P*_TMC_ is 0.477, and *P*_TMR_ is 0.577. Here, *P*_TMR_ is the spin polarization of FM layer, contributing to TMR. The excellent agreement between theory and experiment reveals that the TMC shows a maximum value of 172% at 160 Hz.

### Voltage-induced TMC and TMR

**Figure 3:**
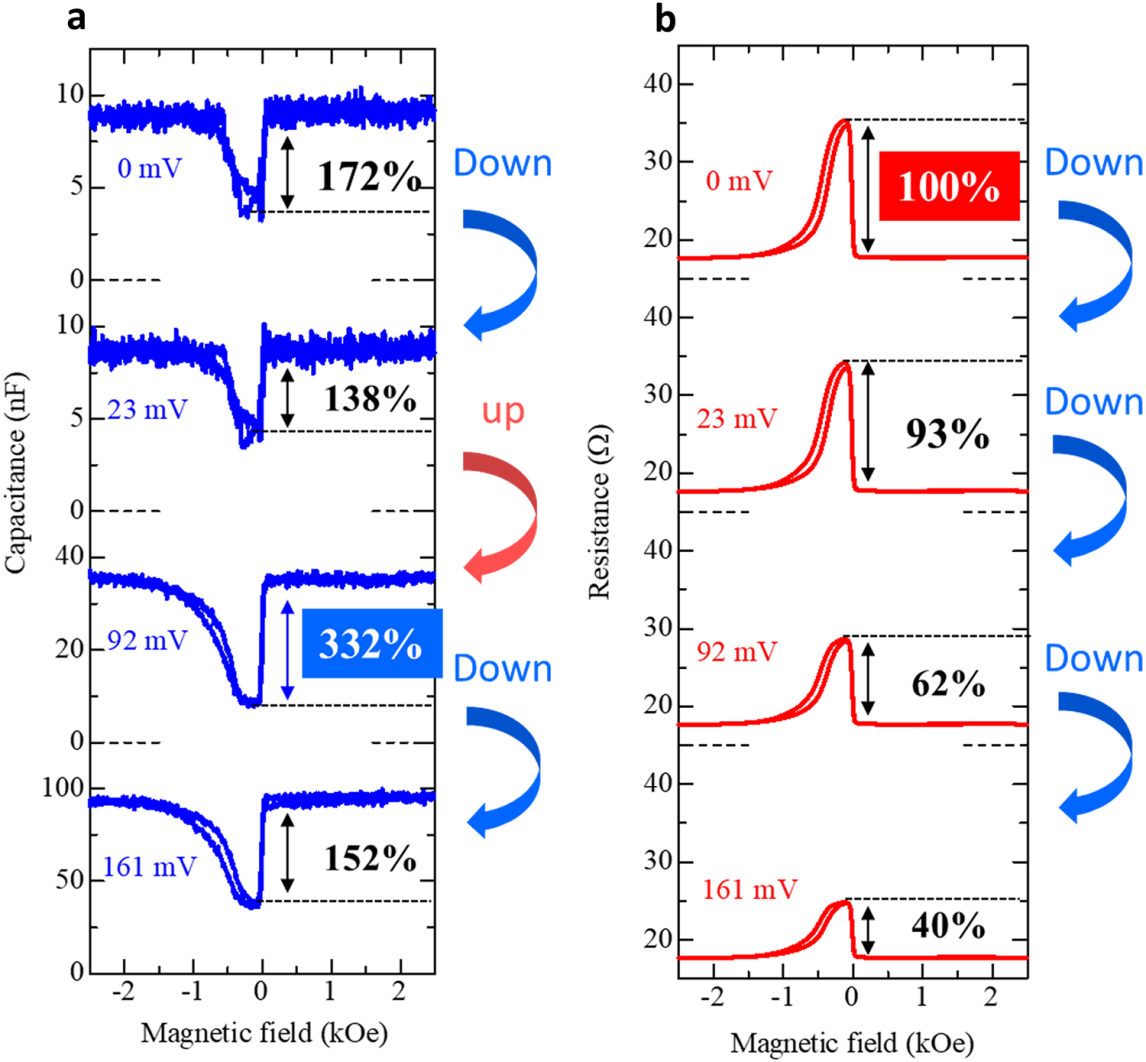
Voltage-induced TMC and TMR curves. (**a**) TMC and (**b**) TMR curves at 160 Hz in an MgO-based MTJ for positive DC bias voltages of 0, 23, 92 and 161 mV. The AC voltage is 2.6 mV_rms_. Note the maximum TMC of 332%, much exceeding the maximum TMR of 100%, and the significant enhancement of TMC, but reduction of TMR, upon biasing.

Figure 3 shows the voltage-induced TMC and TMR curves at 160 Hz. The TMC ratio under the applied bias voltage $V$ at the frequency *f* is defined by $\text{TMC}(f, V) = (C_{{\rm P}}(f, V)-C_{{\rm AP}}(f, V{))}/C_{{\rm AP}}(f, V)$. At around 0 mV, the TMC ratio decreases with the increase of the bias voltage. After that, the TMC ratio increases, and it reaches 332% at 92 mV. A TMC of 332% is the largest value ever reported for MTJs. As increasing the voltage higher than 92 mV, the TMC ratio decreases. On the other hand, TMR rapidly decreases from 100% to 40%. As described in the introduction, the enhancement of $V_{{1/2}}^{{\rm TMR}}$ is important to develop high-performance TMR devices. In our devices, $V_{{1/2}}^{{\rm TMR}}$ is around 138 mV. In contrast, TMC tends to increase with increasing the voltage, and especially at around 92 mV, TMC reaches 332%, which is the double value of TMC near zero bias. This means that $V_{{1/2}}^{{\rm TMC}}$ is not so important in TMC. Instead of this, it is essential to set an appropriate voltage, in which TMC is peaked. One more interesting point is that the noise of the TMC in the high bias region is smaller than that of the TMC in the low bias region. The noise reduction in the high bias voltage is due to the decrease of the effective barrier thickness. The effective MgO thickness decreases with increasing the voltage due to the parabolic shape of potential barrier. Since the capacitance is proportional to the inverse of the thickness, the capacitance increases with increasing the voltage. This behaviour can be easily understood from Fig. 3a. The impedance of the capacitor is expressed by *Z* = 1/*jωC*, where *ω* is the angular frequency. Therefore, the applied voltage reduces the impedance, resulting in a low noise. This fact is consistent with previous works in MTJs, where the noise can be reduced in the high frequency region due to the low impedance^29,39^. From these results, we can realize a large TMC and low noise, i.e. a high signal-to-noise (SN) ratio by setting an appropriate voltage.

We also discuss the scalability of TMC devices. The junction area of fabricated MTJs in this study is 1800 μm^2^. The typical capacitance is about 20 nF (see Fig. 3a). Since the capacitance is proportional to the junction area, for example, the capacitance is 0.1 pF in a junction area of 100 nm × 100 nm. The capacitance is 1 fF in a junction area of 10 nm × 10 nm. These values can be detected using current circuit technology (1 aF is possible to detect). Since the TMR of MTJs used in magnetic read heads or MRAM is about 100%, a TMC of 100% is considered to be necessary in TMC devices. For example, in the case of *C* = 0.1 pF and TMC = 100%, the capacitance *C*_P_ in the P configuration is 0.2 pF and *C*_AP_ in the AP configuration is 0.1 pF. Also, in *C* = 1 fF and TMC = 100%, *C*_P_ is 2 fF and *C*_AP_ is 1 fF. As mentioned above, these values can be detected using current circuit technology. Therefore, TMC heads or memories can work in nano-scale MTJs for readout.

### Bias dependence of voltage-induced TMC

**Figure 4:**
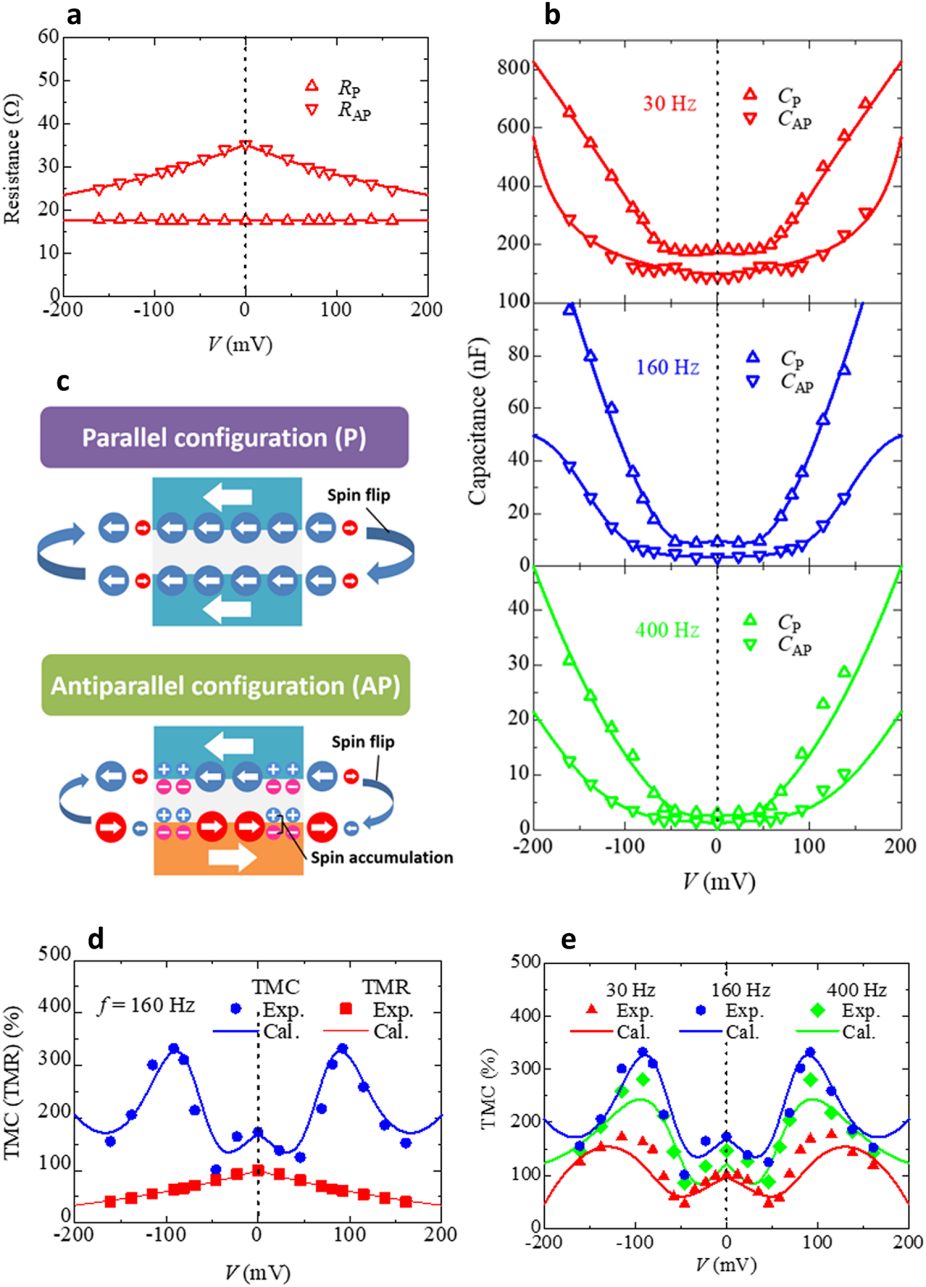
Bias dependence of TMC and TMR. (**a**) DC voltage dependence of the resistance *R*_P(AP)_ in the P(AP) configuration. The calculation of *R*_P(AP)_ is performed using Zhang’s theory, represented by the solid lines. (**b**) DC voltage dependence of the capacitance *C*_P(AP)_ in the P(AP) configuration at 30, 160, and 400 Hz. The *C*_P(AP)_ as represented by the solid lines is calculated using the DF model incorporating the PBA and the Zhang-sigmoid model, as described by Eqs. (1)–(10). (**c**) Schematics of the Zhang-sigmoid model explaining *C*_P(AP)_. (**d**) A comparison of bias dependent TMR and TMC at 160 Hz. (**e**) Bias dependence of TMC at 30, 160 and 400 Hz.

Figure 4 shows the experimental and calculation results of the bias voltage dependence of *R*_P(AP)_, *C*_P(AP)_, TMR and TMC ratio. TMR ratio is calculated using Zhang’s theory^19^. As shown in Fig. 4a, the experimental data of $R_{{\text{P(AP)}}}(V)$ are in good agreement with the calculation using Zhang’s model, where ${K}_{\text{P}\left(\text{AP}\right)}$ is set to 0 (2.48). This means that Zhang’s model is effective for explaining the bias dependence of TMR under both AC and DC model. The calculation of ${C}_{{\rm P(AP)}}(f, V)$ is performed using Eqs. (1)–(10). As shown in Fig. 4b, the measured capacitance $C_{{\rm P(AP)}}(f,V)$ exhibiting a bowl-like behavior is very well described by the theoretical calculations. Here, we used the same parameters of *C*_∞,P(AP)_, *C*_0,P(AP)_, *β*_P(AP)_, *τ*_P(AP)_ and *P*_TMC_ as those used in the investigation of the frequency characteristics of TMC under no bias voltage. The other parameters are shown in Supplementary Table S1 at 160 Hz. The excellent agreement between theory and experiments indicates that the PBA is a good approximation for the expression of the potential-barrier profile in MTJs based on Co_40_Fe_40_B_20_/MgO/Co_40_Fe_40_B_20_ in calculating the bias dependence of *C*_P(AP)_ under AC field. This fact corresponds to the gradual increase of capacitance with applying the voltage due to the decrease of the effective MgO thickness under PBA. The combination of DF model and Zhang-sigmoid theory also proves the validity of spin dynamics of electron–hole dipoles inside the MgO insulator. The Zhang-sigmoid model describes that the spin flip occurs at an applied voltage larger than around *V*_0_ in the sigmoid function. In AP configuration, the spin capacitance appears in the MgO/Co_40_Fe_40_B_20_ interface as shown in Fig. 4c. The presence of the spin capacitance leads to the reduction of the applied voltage inside MgO barrier, where DF-modelled dynamic capacitance is dominant. On the other hand, in P configuration, a sufficiently large voltage is applied inside MgO barrier due to no spin capacitance in the MgO/Co_40_Fe_40_B_20_ interface. Therefore, the capacitance in P configuration rapidly increases at around *V*_0,P_, which is smaller than *V*_0,AP_. The difference between *V*_0,P_ and *V*_0,AP_ causes a large voltage-induced TMC, reaching 332%. This means that the voltage to bring about spin flip is quite different in P and AP configurations, respectively, that is, the energy that electrons acquire for spin flip differs from each other in P or AP, respectively. The threshold voltage *V*_0,P(AP)_ can be described by Zhang-sigmoid model.

Figure 4d shows the bias dependence of TMC and TMR at 160 Hz. As shown in Fig. 3a already, TMC decreases at around zero bias, and then it has a maximum value of 332% at 92 mV. This tendency can be observed in the forward and reverse bias region. It is also noted that TMC ratio is larger than TMR ratio in the entire bias region. This means that TMC is superior to TMR by setting an appropriate frequency for practical use. Figure 4e shows the bias dependence of TMC ratio at 30, 160 and 400 Hz. In the frequency ranging from 30 to 400 Hz, the same tendency can be seen in.

TMC-*V* curves, and TMC is larger than TMR at any bias voltage. As can be seen in this figure, the maximum TMC is 332% at 160 Hz with the application of 92 mV. We emphasize that the TMC of 332% is the highest value ever reported for MTJs. The solid lines in Fig. 4e represent calculation results. The fitting parameters are listed in Supplementary Table S1. Here, *C*_∞,P(AP)_, *C*_0,P(AP)_, *β*_P(AP)_, and *τ*_P(AP)_ are the same parameters in fitting the frequency dependence of TMC under no bias voltage, shown in Fig. 2c. The other parameters, appeared in Zhang-sigmoid, PBA, and SDD models, are newly fitted to experimental data. As one can see, there is an excellent agreement between theory and experiment. The detailed results on the frequency dependence of TMC under bias voltage are shown in Supplementary Fig. S1.

### Prediction of an extremely large voltage-induced TMC

**Figure 5:**
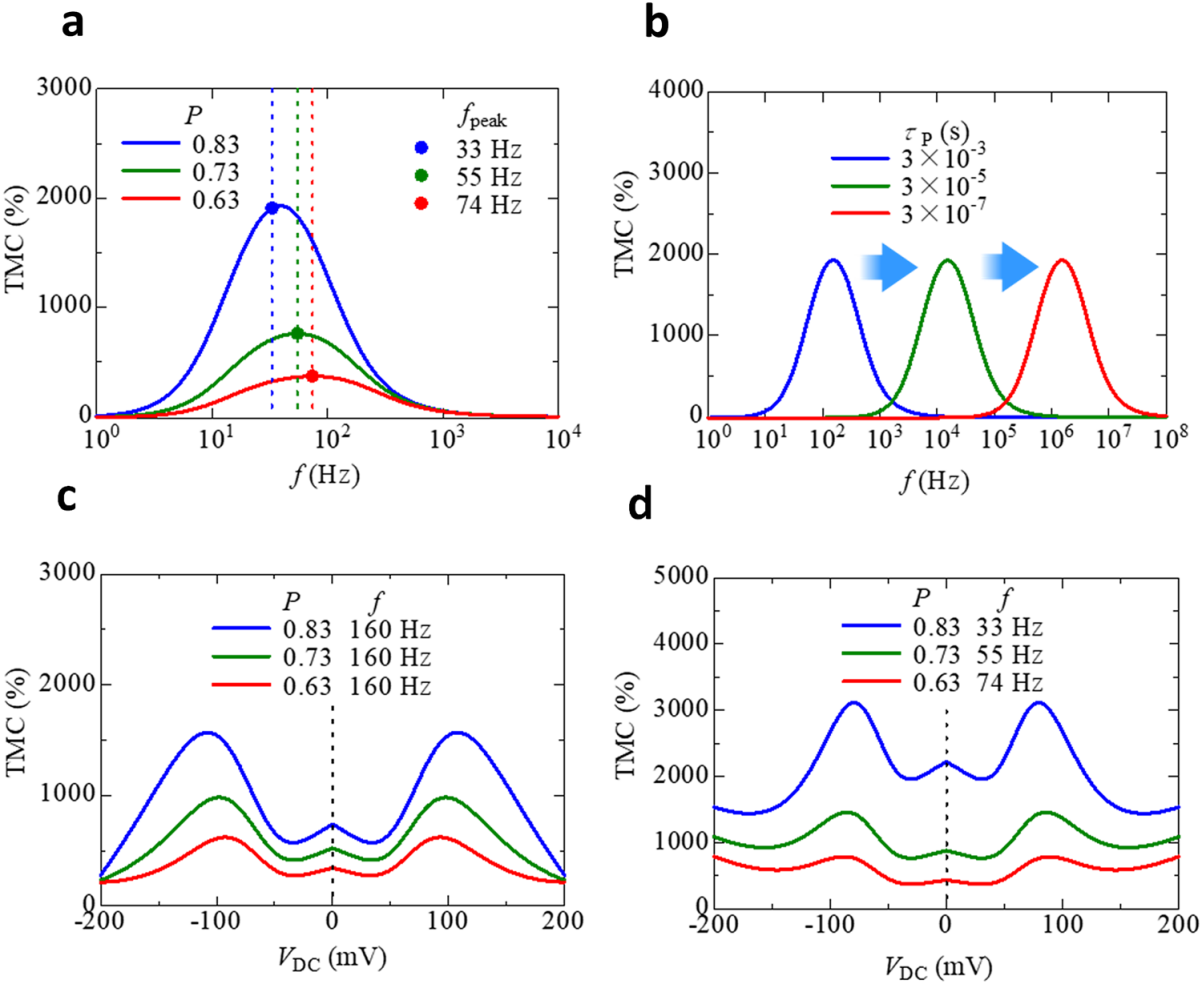
Prediction of an extremely large voltage-induced TMC. (**a**) Calculated frequency dependence of the TMC under no bias voltage with varying spin polarization *P*. (**b**) Calculated frequency dependence of the TMC with varying *τ*_P_. The peak position of the TMC is shifted to a high frequency region for a short *τ*_P_. Calculated voltage-induced TMC with varying (**c**) *P *at 160 Hz and (**d**) at *f*_peak_. Note the maximum TMC is about 3000%.

Finally, we show the prediction of an extremely large TMC. Figure 5a shows the calculated frequency dependence of the TMC at a zero bias voltage with varying spin polarization *P*. The assumed maximum value of *P* is 0.83, which is estimated experimentally for high-performance MgO-based MTJs at room temperature^40^. The parameters used in the calculation of the TMC under no bias voltage are *C*_∞,P(AP)_ = 0.80 (0.90) nF, *C*_0,P(AP)_ = 1037 (1221) nF, *β*_P(AP)_ = 0.986 (0.999) and *τ*_P_ = 0.0118 s. As can be seen from Fig. 5a, the maximum TMC increases from 363% to 1906% with increasing *P* from 0.63 to 0.83. The *f*_peak_, at which the TMC ratio is peaked at maximum, is 33, 55 and 74 Hz, respectively. Figure 5b shows the calculated frequency dependence of the TMC under no bias voltage with varying *τ*_P_. The maximum peak of the TMC is shifted to a high frequency region on the order of MHz for a short *τ*_P_ in the sub-μs scale. The DF model suggests that the relaxation time is determined by the oscillation speed of electric dipoles formed near the FM/insulator interfaces. The relaxation time is short in a high oscillation speed. For a short relaxation time, the thickness of the insulator should be thinner. Therefore, the formation of a thinner MgO layer is necessary for a high frequency operation. In fact, the recent paper demonstrates a high frequency operation of ~ 100 MHz, corresponding to a relaxation time of sub ns, in FeCo-MgF nanogranular system^41^. Figure 5c, d shows the calculated voltage-induced TMC with varying *P* at 160 Hz and *f*_peak_, respectively. The parameters used in the calculation of the bias dependence of the voltage-induced TMC are *κ* = 0.1, *S* = 1800 μm^2^, *λ* = 0.1 nm. $\phi$_0,P(AP)_ is 2.0 (0.046) eV, *n*_0,AP_ is 0.354 × 10^23^ cm^−3^, *K*_0,P(AP)_ = 26.7 (22.4) V^−1^, *V*_0,P(AP)_ = 0.047 (0.085) V, and *β*_P(AP)_ = 64.7 (29.8). The maximum voltage-induced TMC increases from 788% to 3114% with increasing *P* from 0.63 to 0.83. The prospect of achieving TMC of more than 3000% is attractive for the development of spintronics. It will have profound impact on spin-based circuit designs, non-volatile memories, magnetic sensing, spin logic devices, and voltage controlled electronic components. Traditionally, variable capacitors are bulky, mechanical, or narrowly ranged. Comparatively, the MTJs as capacitors are small in size, broadly ranged under the voltage control, and fully electronic and non-mechanical.

In summary, we have successfully observed a large TMC effect in MgO-based MTJs at room temperature. The voltage-induced TMC increases up to 332%, which is the largest value ever reported for any MTJs. We have understood the full mechanism of this dramatic effect both qualitatively and quantitatively by our explained newly proposed DF model incorporating PBA, SDD, and the Zhang-sigmoid model. This calculation predicts that the voltage-induced TMC could potentially reach 3114% in MTJs with a spin polarization of 83%. Our theoretical and experimental findings provide new insights into the exact mechanism of the voltage-induced AC spin transports in MTJs. The works will produce a new platform for the development of spintronics applications and electrical engineering.

## Methods

### Preparation of the samples

The MTJs were prepared by using a home-made high vacuum magnetron sputtering system with a base pressure of 2 × 10^–8^ Torr. The MTJs have the following layer sequence: SiO_2_/Ta(5)/Co_50_Fe_50_(2)/IrMn(15)/Co_50_Fe_50_(2)/Ru(0.8)/Co_40_Fe_40_B_20_(3)/MgO(2)/Co_40_Fe_40_B_20_(3)/Contact layer (thickness in nm). We deposited all the metallic layers in DC mode under a sputtering Ar gas pressure of 1.5 mTorr, and the MgO layer deposited with radio frequency (RF) magnetron sputtering under an Ar gas pressure of 1.1 mTorr. Using standard photolithography, we have patterned the multilayer MTJ stacks into a junction area of 1800 μm^2^ with an elliptical shape with Ar ion-milling and SiO_2_ insulation overlayer. Afterwards, we annealed the MTJs at 310 °C for 4 h in a vacuum of 10^–6^ Torr under magnetic field of about 4.5 kOe to define the pinning axis for the bottom Co_40_Fe_40_B_20_ ferromagnetic electrode.

### Measurements of the voltage-induced TMC

The frequency characteristics and the bias voltage dependence of the TMC and TMR for MTJs were measured by an AC four-probe method using an Agilent Technologies 4284A LCR meter at room temperature. The frequency ranged from 50 Hz to 1 MHz and the bipolar bias voltage was applied up to 200 mV. The AC voltage was set to 2.6 mV_rms_. The magnetic field was applied along the magnetic easy-axis direction to 1.4 kOe.

## Supplementary Information

Supplementary Information.
