# Supplementary material for: Observation and theoretical calculations of voltage-induced large magnetocapacitance beyond 330% in MgO-based magnetic tunnel junctions
Source: Sci Rep. 2021 Jul 12;11:13807. doi: 10.1038/s41598-021-93226-4 (PMC8275788; doi:10.1038/s41598-021-93226-4)
Supplement: Supplementary file 1 — Supplementary Information. [file 41598_2021_93226_MOESM1_ESM.pdf]

Observation and theoretical calculations of voltage-induced large magnetocapacitance beyond 330% in MgO-based magnetic tunnel junctions

---

Supplementary Information

Kentaro Ogata[1], Yusuke Nakayama[1], Gang Xiao[2] and Hideo Kaiju[1,3]

[1]Faculty of Science and Technology, Keio University, Yokohama, Kanagawa 223-8522, Japan

[2]Department of Physics, Brown University, Providence, RI 02912, USA

[3]Center for Spintronics Research Network, Keio University, Yokohama, Kanagawa 223-8522, Japan

Correspondence and requests for materials should be addressed to H. K. (email: kaiju@appi.keio.ac.jp).

In this Supplementary Information section, we present detailed results on the frequency characteristics of voltage-induced TMC, parameters used in our calculations, the calculation results with and without PBA, and comparison between MTJs in this study and previous ones.

Frequency characteristics of TMC under the bias voltage. Figure S1 shows the frequency dependence of TMC under bias voltage of 88, 92, 115 and 161 mV. The TMC is peaked at a specific frequency. Interestingly, the TMC is very sensitive to the bias voltage. As a result of the detailed investigation, it is found that the TMC ratio shows a maximum value of 332% at 160 Hz at 92 mV.

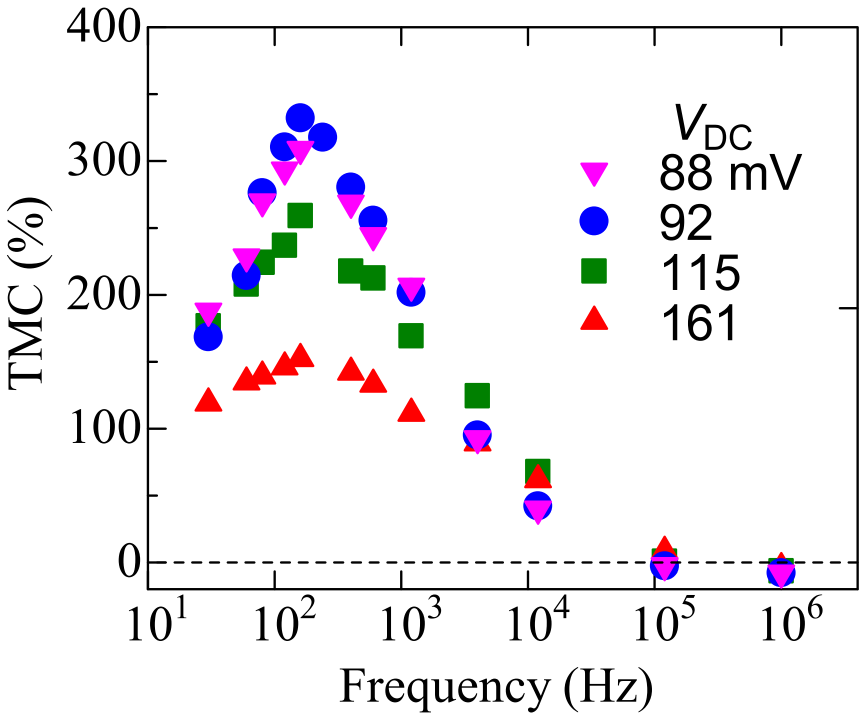

Figure S1 | Frequency characteristics of voltage-induced TMC. Frequency dependence of TMC ratio under bias voltage.

Parameters used in the calculation. Table S1 shows the detailed parameters used in the calculation of voltage-induced TMC in Figs. 4b, 4d, and 4e.

Table S1 Parameters used in the calculation of voltage-induced TMC of Fig. 4b, 4d, and 4e.

f (Hz)	$C_{\infty,P(AP)}$ (nF)	$C_{0,P(AP)}$ (nF)	$\beta_{P(AP)}$	P	τ_P (s)	$\phi_{0,P(AP)}$ (eV)
30	0.80(0.90)	1037(1221)	0.986(0.999)	0.477	0.0118	0.168(0.0275)
160	0.80(0.90)	1037(1221)	0.986(0.999)	0.477	0.0118	2.00(0.0460)
400	0.80(0.90)	1037(1221)	0.986(0.999)	0.477	0.0118	0.841(0.0582)

f (Hz)	$V_{0,P(AP)}$	$n_{0,P(AP)}$ (cm^{-3})	$K_{P(AP)}$ (V^{-1})	$\alpha_{P(AP)}$	γ
30	0.059(0.108)	0.792	13.0(0.0)	65.2(17.6)	0.1
160	0.047(0.085)	0.354	26.7(22.4)	64.7(29.8)	0.1
400	0.036(0.053)	0.300	33.1(23.8)	79.3(20.6)	0.1

Calculation results with and without PBA. Figure S2 shows the fitting results for a model with and without PBA assumption. It is found that the experimental data provide an excellent fit to the calculation results with PBA, whereas they do not fit well with calculation results without PBA.

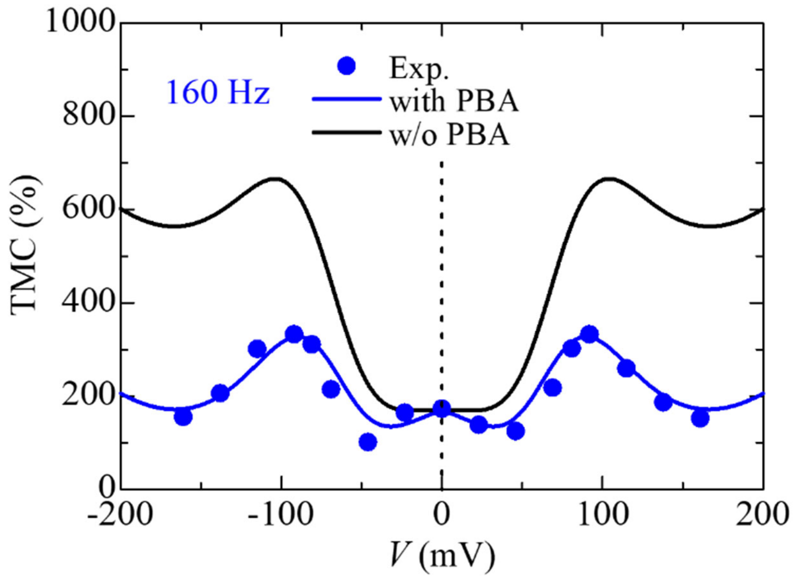

Figure S2 | Fitting results on TMC-V for a model with and without PBA assumption.

Comparison between MTJs in this study and previous ones. In this study, we fabricate a lot of MTJ samples on Si wafer. The thickness of MgO is slightly different depending on the position of samples on this wafer. The slight change in thickness give a large influence on TMC properties. In MTJ samples, reported in our previous paper (*Sci. Rep.* **8**, 14709 (2018)), TMC ratio was about 100%. Also, the breakdown occurred at voltages lower than spin-flip voltage V_0. As a result, we could not observe a large TMC. On the other hand, after that, as a result of the measurement of TMC in other MTJ samples, we observed a large TMC, showing in our paper, and obtained a large spin-flip voltage.
